# Potential of *Metschnikowia* yeasts in green applications: a review

**DOI:** 10.3389/fmicb.2025.1652494

**Published:** 2025-10-02

**Authors:** Jiayue Liu, Anna Rygała, Bolin Zhang, Dorota Kręgiel

**Affiliations:** ^1^Interdisciplinary Doctoral School, Lodz University of Technology, Łódź, Poland; ^2^Department of Environmental Biotechnology, Lodz University of Technology, Łódź, Poland; ^3^Department of Biological Sciences and Technology, Beijing Forestry University, Beijing, China

**Keywords:** *Metschnikowia*, green technology, agro-industrial waste, benefits, limitations

## Abstract

The unconventional yeasts *Metschnikowia* spp. represent a valuable microorganisms with enormous yet untapped potential. *Metschnikowia* species are briefly reviewed, demonstrating that taxonomic and genomic analysis can open numerous opportunities to exploit their unique character and potential in the development of modern winemaking and brewing, probiotics and biocontrol, and the synthesis of single-cell proteins. These yeasts can be used in both bioprocesses and biorefineries, contributing to the production of biofuels and unique products recovered from agro-industrial wastes. This review, through a comprehensive bibliographic analysis, examines various green strategies for the production of alcohols, lipids, unsaturated fatty acids, and other valuable metabolites. Furthermore, the article discusses the challenges and barriers hindering the full implementation of *Metschnikowia* spp. in new approaches and technologies.

## 1 Introduction

**Figure 1:**
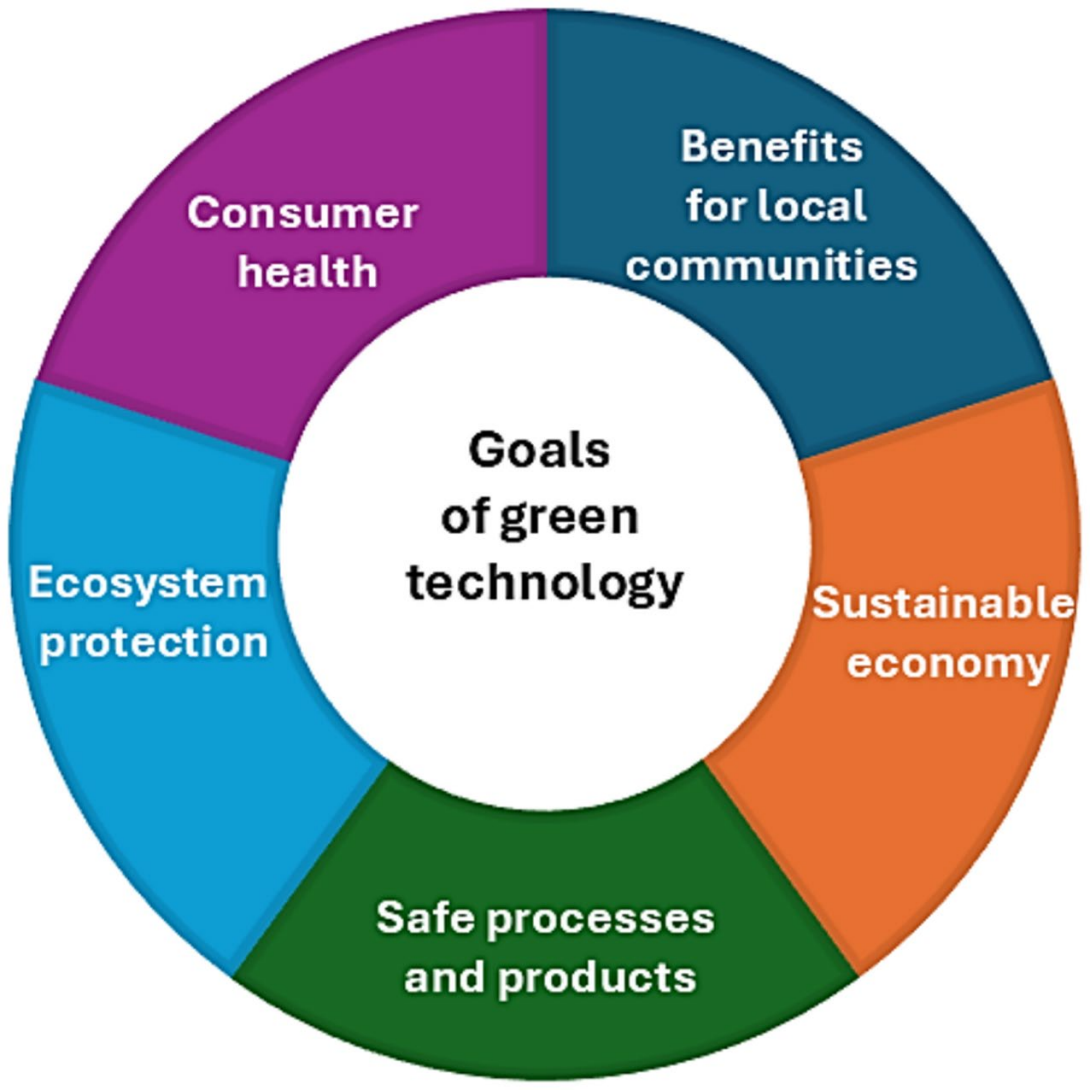
The main goals of green technology.

Green technology is commonly defined as the development and use of processes that minimize the negative impact of human activity on the environment and society (Figure 1).

It encompasses a diverse range of technologies and practices that address environmental issues, paving the way for sustainable development. It creates solutions and strategies to mitigate the effects of climate change, reduce environmental degradation, and promote the efficient use of natural resources. Green technology leverages both scientific knowledge and innovation to conserve natural resources, mitigate greenhouse gas emissions, and promote a circular economy. A key aspect of green technology is also its positive contribution to human health, both through the development of processes that do not negatively impact the health of workers involved in production and through the nature of manufactured consumer goods that support consumer health (Al-Emran and Griffy-Brown, 2023).

Among the main principles of green technology, including renewable energy and energy efficiency, transportation, water and wastewater treatment, and carbon capture technologies, agro-industrial waste management also plays a crucial role. Waste management technologies include advanced recycling plants that convert waste into valuable resources. Sustainable agricultural practices, on the other hand, aim to reduce the environmental impact of food production and maintain food security (Domínguez et al., 2024; Mishra et al., 2023).

The idea of a “circular economy,” which refers to the use of organic waste from one industry as a raw material for another, is based on the sustainability principle known as the “5Rs” (reduction, recycling, reuse, recovery, and regeneration) and replaces the traditional linear model (production, use, disposal). In recent decades, the growth of the food and agro-industrial sectors has dramatically increased food waste production. The amount of waste generated by agro-based industries has more than tripled (Bibi et al., 2023).

**Figure 2:**
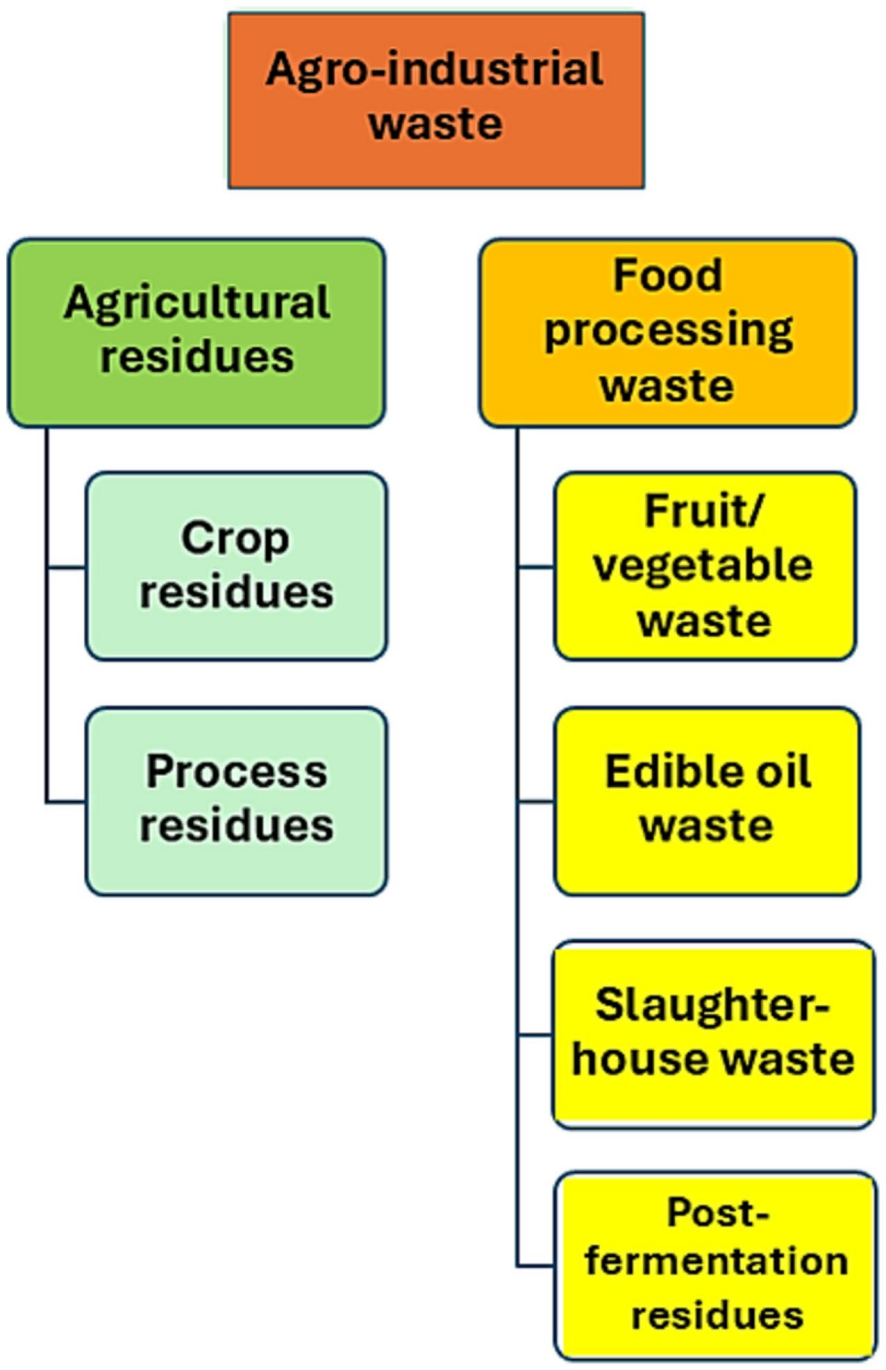
Different types of agro-industrial waste.

The Food and Agriculture Organization of the United Nations (FAO) estimates that approximately 1.3 billion tons of food are wasted each year, representing one-third of global production (Food and Agriculture Organization of the United Nations, 2022). Wastes pollute the environment. However, they serve as beneficial biomass resources. In addition to food waste, various types of agro-industrial residues are generated annually worldwide (Figure 2). Such organic wastes, a rich source of carbohydrates, proteins, lipids, organic acids, and other essential compounds, can be used in bioconversion processes (Sharma et al., 2022). They can serve as an inexpensive substrate for the manufacturing of various goods, including biogas, biofuel, single-cell biomass, as well as probiotics, biocontrol agents, fertilizers, enzymes, vitamin supplements, or antioxidants (Bibi et al., 2023).

The production of high-quality products represents one possibility for exploiting rich and valuable sources of organic molecules derived from agricultural waste and beneficial microorganisms (Phiri et al., 2024).

## 2 Unveiling *Metschnikowia* spp.: biology, physiology, and taxonomic studies

Several types of yeasts, which are eukaryotic microorganisms, have been widely used in various industries due to their potential applications, both for fermentation and the production of specific metabolites. *Metschnikowia* spp. are non-conventional yeasts with great biotechnological potential, and knowledge about them is still relatively limited, in comparison to conventional *Saccharomyces cerevisiae*.

The *Metschnikowia* genus was first identified approximately 130 years ago, and currently, the number of described *Metschnikowia* species exceeds 80 (Vicente et al., 2020). Species of the genus *Metschnikowia* form a monophyletic group within the family Metschnikowiaceae, which also includes the several other genera, such as *Australozyma, Candidiozyma, Clavispora, Danielia, Gabaldonia, Gaillardinia, Helenozyma, Hermanozyma, Isabelozyma, Osmozyma, Soucietia, Sungouiella, Tanozyma*, or *Wilhelminamyces* (Liu et al., 2024; Index Fungorum, 2025; NCBI Taxonomy Browser, n.d.).

The principal habitats where *Metschnikowia* species are encountered regularly include various parts of plants, namely flowers, fruits, barks, and leaves, or also the digestive tract or frass of some insects, as well as aquatic organisms (Kurtzman et al., 2018; Agarbati et al., 2024a). It is worth noting that some species of the *Metschnikowia* genus are globally distributed, while others exhibit extreme endemism. The nature of the association between *Metschnikowia* species and insects remains unclear; however, the relationship seems to be species-specific (Lachance, 2016).

The ecological diversity of *Metschnikowia* yeasts is not the only characteristic of these unique yeasts. Their uniqueness also extends to their morphology and physiology. *Metschnikowia* sp. reproduces by multilateral budding. The cells are spherical or ellipsoidal and may be pear-shaped, cylindrical, or lunate in shape. Pseudohyphae are weakly developed but often absent. In sexual reproduction, the ascospores are needle-shaped, tapered at one or both ends, sometimes swollen along one half, and the asci are elongated, club-shaped, spheroid, or ellipsoid-stalked. Depending on the species, one or two spores are produced per ascus. In some cases, spores can reach enormous sizes, exceeding 200 μm in length. In some strains, ascospore formation is preceded by the development of chlamydospores. *Metschnikowia* species also differ in the efficiency of forming these thick-walled cells. The adaptive properties of these features remain to be elucidated (Lachance, 2011; Lachance, 2016). Some species of this genus can produce pulcherrimin, a red pigment constituting a chelate of pulcherrimic acid and iron ions (Sipiczki, 2020). *M. pulcherrima* species can express different extracellular hydrolytic enzymes, namely amylase, cellulase, glucanase, β-glucosidase, β-lyase, lipase, lichenase, pectinase, protease, sulfite reductase, and xylanase, which makes them very interesting microorganisms in bioconversion processes (Mateo, 2023; Chaudhary and Karita, 2017).

The diversity of *Metschnikowia* strains also extends to their molecular characteristics. The yeast identification is usually based on the assumption that differences in barcodes are more minor within a single species than between species. The most commonly used barcodes are chromosomal repeat segments encoding ribosomal RNA. Molecular analysis of such segments in strains of several species belonging to *Metschnikowia*, conducted by Sipiczki et al. (2024), showed that this is not possible for the species of this genus. In these studies, intragenomic diversity significantly exceeded the threshold gaps used to differentiate related yeast species. The genome structures of various *Metschnikowia* spp. isolates were compared using RAPD and RFLP of mitochondrial DNA, demonstrating their high heterogeneity. Also, the sequence analysis of the *PUL4* gene (a component of the *PUL* cluster) involved in pulcherrimin production revealed substantial intragenomic differences, suggesting that the genomes may be chimerized. These features make *Metschnikowia* spp. unique among yeasts and indicate that these traits and features evolve in a non-standard manner. When the molecular differences were compared with the phenotypic differences, no clear correlation was observed between the examined genetic/genomic diversities and the phenotypic diversities. Thus, according to Sipiczki and co-workers, none of the molecular tests can be used for differentiating strains that exhibit different phenotypes (Sipiczki, 2022; Sipiczki et al., 2024).

Recent phylogenetic, genetic, and genomic studies at the molecular level have raised questions about the taxonomic classification of species within the *Metschnikowia* genus. These data, combined with the results obtained in many studies through comprehensive analysis of primary and secondary barcode sequences, physiological features, and hybridization experiments, prove that the species within *Metschnikowia* cannot be distinguished from each other based on any of the phenotypic, phylogenetic, or biological concepts. These taxonomic properties were further supported by Troiano et al. (2023), who conducted a comparative genomic analysis of seven strains belonging to the *M. pulcherrima* clade and revealed the absence of single-copy markers for species differentiation. Sipiczki proposed combining species belonging to the *M. pulcherrima* clade, characterized by the formation of pulcherrimin (*M. andauensis, M. fructicola, M. leonuri, M. pulcherrima, M. rubicola, M shanxiensis, M. sinensis,* and *M. zizyphicola*), into a single species under the oldest species name, *M. pulcherrima* (Sipiczki, 2022; Sipiczki and Czentye, 2024).

## 3 Safety aspects

Molecular biology and advanced genetic techniques have become essential tools in various fields of interest, including taxonomy, identification, classification, and metabolite production, as well as in potential applications. However, the safety of yeast used in biotechnological processes is also crucial to ensure benefits for humans and the environment (Tullio, 2022).

Knowledge about microorganisms involved in biotechnological processes has increased over the past decades, and the rapid development of molecular biology techniques has allowed for a deeper understanding of the genetic basis and specific metabolic pathways of microorganisms involved in these processes. However, the genetic refinement of microbial strains involved in processing remains controversial. Genetically modified microorganisms (GMOs) still encounter disapproval and are subject to extensive regulatory requirements. The use of GMOs as cell factories in closed systems that prevent their release into the environment is the least problematic aspect. Still, in the presented idea of green technologies, it seems to be wholly excluded (Plavec and Berlec, 2020).

Genome sequences are keystone data to explore the applicability of *Metschnikowia* spp. bioresources. They are also crucial for a comprehensive safety assessment, which is the principal regulatory concern, as requested by the European Food Safety Authority (EFSA) (EFSA, 2018; Binati et al., 2021; Miguel et al., 2022).

The safety analysis of yeasts is still under consideration because protocols are less developed than for bacterial assessments, and standardized methods have not yet been developed. According to EFSA, genome sequences should be searched to identify the presence/absence of metabolic pathways involved in toxigenicity or antifungal drug resistance. If detected, appropriate analysis is required to validate *in silico* evidence (EFSA, 2018). Genome studies are necessary because drug-resistant fungal infections pose a growing global health threat. However, no databases have been developed specifically for detecting antifungal drug resistance genes. In the studies of Larini et al. (2025), none of the *Metschnikowia* strains were capable of producing biogenic amines. In a recent study conducted by Rahmat et al. (2024), the toxicity assessment of extracts of *M. pulcherrima* (formerly *M. persimmonsis*) strains showed that they had no harmful effects on the liver and mitochondria of zebrafish, and no potential risk of cardiotoxicity was observed. Furthermore, other strains of *M. pulcherrima* (formerly *M. ziziphicola*), selected as potential probiotics, did not show any hemolytic activity (Staniszewski and Kordowska-Wiater, 2023).

However, the safety data are not consistent with previous publications that described a single strain of *M. pulcherrima* causing disease in immunocompromised patients (Mohl et al., 1998). A case report later described a non-pigment-producing strain of *Metschnikowia* isolated from the skin of a patient with dermatitis (Kuan et al., 2016). Another case involved an atypical strain of *Metschnikowia* isolated from a patient with leukemia (Savini et al., 2013).

In this context, the issue of the significant genetic diversity of *Metschnikowia* strains, which may reflect certain features typical of pathogenic microorganisms, is still the subject of intensive research. For example, the whole genome sequence of the pathogenic *M. bicuspidata* LNES0119 strain, responsible for disease effects in the crab *Eriocheir sinensis,* was sequenced by Jiang and co-authors (Jiang et al., 2022). Within the 16.13 Mb genome of *M. bicuspidata* LNES0119, encoding 5,567 genes, 1,467 genes were identified with significant homology to genes from the pathogen-host interaction database. Comparative genomic analyses of three *M. bicuspidata* strains and one nonpathogenic *M. pulcherrima* strain revealed 331 unique genes in *M. bicuspidata* LNES0119, 30 of which were putatively associated with its pathogenicity. Genomic and comparative analyses showed that the genome of *M. bicuspidata* LNES0119 contains a variety of putative pathogenic genes, primarily involved in cell wall assembly and construction. These genes may play a crucial role in adapting to the host environment, acting as virulence factors in pathogenicity, or triggering a cell-mediated host immune response.

In addition, special attention was paid to the genome analysis of *Candida lusitaniae*, a pathogenic yeast responsible for candidemia in humans and most phylogenetically related to *Metschnikowia* in the so-called “GTC clade.” Phenotypic echinocandin resistance in *C. lusitaniae* results from a missense mutation (S645F) in the *FKS1* gene, which was not found in *Metschnikowia* strains. Specifically, sequenced *M. pulcherrima* strains exhibited more than one copy of the *FKS1* protein-encoding gene, which contained either serine at position 645 (similar to the nonpathogenic wild-type *C. lusitaniae*) or proline (Larini et al., 2025).

Formally, *M. pulcherrima* was included on the list of microorganisms approved for food use developed in a joint project by the International Dairy Federation (IDF) and the European Food and Feed Cultures Association (EFFCA) in 2002. Later additions suggested by the IDF National Committees and EFFCA members, as well as additions found through a scientific literature search, were also included on the list (Bourdichon et al., 2012).

In the United States, two *Metschnikowia* species, *M. pulcherrima* and *M. fructicola*, have been Generally Recognized As Safe (GRAS) by the Food and Drug Administration (FDA) (FDA, 2022). Under Sections 201 and 409 of the Federal Food, Drug, and Cosmetic Act, any substance that is intentionally added to food is subject to premarket review and approval by FDA, unless the substance has been generally recognized to be safe by qualified experts under the conditions of its intended use, or unless the use of the substance has been otherwise excepted from the definition of a food additive. Therefore, GRAS is not simply a special regulatory marker. It represents a comprehensive approach to ensuring that food and dietary products are safe for their intended purposes.

In the European Union, EFSA has published a peer review risk assessment of the use of the active substance *M. fructicola* NRRL Y-27328 as a pesticide (EFSA, 2017). A completely natural origin characterizes this strain - it has been isolated from grapes grown in central Israel. The positive opinion was reached based on the evaluation of the representative uses of *M. fructicola* NRRL Y-27328 as a fungicide on stone fruits, strawberries, and grapes. Regulation (EU) 2018/1915 of 6 December 2018 approved the active substance *M. fructicola* strain NRRL Y-27328 under Regulation (EC) No 1107/2009 of the European Parliament and of the Council, concerning the placing of plant protection products on the market (European Union, 2018).

The results of molecular studies may provide a novel resource of knowledge for further analysis of the pathogenic mechanisms in *Metschnikowia*, as well as for the identification of potential targets for further research and therapeutic intervention.

According to the last EFSA statement, microorganisms used in the food chain, either as active agents, biomasses, or as production organisms of substances of interest, should be subject to a premarket authorization process. This procedure includes a complete molecular characterization of the organism under assessment. Data analysis can provide information on the unambiguous taxonomic identification of the strains, on the presence of genes of concern (e.g., those encoding virulence factors, resistance to antimicrobials of clinical relevance for humans and animals, production of harmful metabolites, or of clinically relevant antimicrobials) and on the characterization of genetic modifications (EFSA, 2024).

Therefore, the application potential of the strain must be verified through extensive studies to exclude the presence of any potential pathogenic features.

## 4 Application potential

The unique features of yeasts of the genus *Metschnikowia* provide potential opportunities for their use in many biotechnological processes as well as in enology, agriculture, and the food and cosmetics industries (Haniffadli et al., 2024). The potential use of these yeasts in biocatalysis is particularly interesting. Research results are available on the production, from precisely defined substrates, various metabolites such as ethanol (Chaudhary and Karita, 2017) and lipids (Santamauro et al., 2014). What is more, *Metschnikowia* spp. demonstrate the potential to produce biodegradable surfactants (Kumari et al., 2021), low-calorie sweeteners (Sasahara and Izumori, 2005), as well as analytical reagents (Meena et al., 2014). The list of selected metabolites of the yeast *Metschnikowia* spp. from precisely defined substrates is presented in Table 1. It is complemented by the possible uses of these yeasts in the cosmetics industry, winemaking, and brewing.

**Table 1:** Possible biotechnological applications of *Metschnikowia* species.

Application potential	Main substrate(s)	Product(s)	*Metschnikowia* species	Origin	References
Low-calorie sweeter	L-psicose	L-talitol	*M. koreensis* LA1	Isolate from the soy sauce mash	Sasahara and Izumori (2005)
Biofuel	Glycerol	Lipids	*M. pulchererima*	No data	Santamauro et al. (2014)
	Glucose	Ethanol	*M. cibodasensis* Y34	Isolate from *Abelia* flowers	Chaudhary and Karita (2017)
Biodegradable surfactant	Glucose	Sophorolipid	*M. churdharensis* CIG-6A^T^	Isolate from the intestine of a stingless bee	Kumari et al. (2021)
Analytical reagents	Racemic aryl secondary alcohols and 1,2-diols	Single enantiomer	*M. koreensis* MTCC-5520	Isolate from flowers in Korea (Hong et al., 2001)	Meena et al. (2014)
Winemaking	Verdicchio grape juice	Biocontrol and wine aroma enhancing	*M. pulcherrima* DiSVA 269	Isolate from the winery	Canonico et al. (2023)
	Aglianico grape must	Aglianico wine with improved volatile profiles	*M. fructicola* ST1	Isolate from Aglianico grapes and musts (Sorrentino et al., 2012)	Boscaino et al. (2019)
	Verdejo grapes	Verdejo white wine with a lower alcohol level and fresher aroma	*M. pulcherrima* NS-EM-34	Pre-commercial strain (no-data)	Ruiz et al. (2018)
	Grape juice Chardonnay must	Bioprotection under winemaking conditions with *S. cerevisiae*	*M. cerradonensis*, *M. koreensis, M. kunwiensis, M. peoriensis, M. pulcherrima, M. reukaufii*	isolates from flowers, fruit juice, and musts; commercial strains (Lallemand)	Aragno et al. (2024)
	Grape juice Chardonnay and Shiraz musts	Fermentation with *S. cerevisiae* and *S. uvarum* resulted in positive sensory characteristics	*Metschnikowia pulcherrima* AWRI1149	Collection strain Australian Wine Research Institute (AWRI) Wine Microorganism Culture Collection (WMCC)	Varela et al. (2016)
	Grape must	Catarratto wine with better preservation of aromatic compounds and color, and a positive impact on the oxidative stability of wine	*M. pulcherrima* MP346	Commercial strain Flavia® (Lallemand)	Naselli et al. (2023)
	White grape juice	Low alcohol wines	*M. pulcherrima* MP1-MP6	Isolates from uninoculated Shiraz fermentation (South Australia)	Hranilovic et al. (2020)
	Cabernet Sauvignon grape musts	Increasing intensity of desirable sensory attributes	*M. pulcherrima* AWRI1149, AWRI3050	Commercial dry yeasts (AB Biotek)	Varela et al. (2021)
	Airén grape must	Increasing acidity and modifying the volatile profile of wine	*M. pulcherrima* M29	Commercial strain (Lallemand Bio)	Escott et al. (2022)
	Garganega grape must	Protection of grapes against fungal infections, wine with distinguished aromatic characteristics	*M. pulcherrima* Level^2^ Initia	Commercial strain (Lallemand)	Binati et al. (2023)
	Chardonnay grape must	Chardonnay white wine protection, good sensory characteristics	*M. pulcherrima* MCR24	Commercial dry yeast (AEB France)	Lebleux et al. (2023)
	Apple musts	Apple cider with acidity modulation, aroma enhancement, and color improvement	*M. koreensis* *M. reukaufii* *M. pulcherrima*	Isolates from naturally fermented cider	Wu et al. (2025)
	Grape must	Muscat wine with improved quality	*M. pulcherrima* Mp0520	Isolate from the Muscat Hamburg grape	Guo et al. (2025)
	Apple must Apple/chokeberry must	Chemical complexity of apple wines	*M. pulcherrima* NCYC 747, *M. sinensis* LOCK1143	Collection strain (NCYC, UK), isolate from strawberry fruits (LOCK, Poland)	Kregiel et al. (2022b)
	Honey wort	Mead with lower ethanol content, higher glycerol level, and rich volatilomes	*M. pulcherrima* NCYC 747	Collection strain (NCYC, UK)	Kregiel et al. (2025)
Brewing	Brewery wort	Beer with an alcohol content of <0.5%	*M. pulcherrima* MP1-MP9	Isolates from Polish grapes	Klimczak et al. (2024)
		American IPA-style beer	*M. pulcherrima* 62, 82, 86	Isolates from Albanian grapes	Karaulli et al. (2024)
Cosmetic ingredients	*M. agaves* cells	Hydrolyzate with α-glucan oligosaccharides and β-glucan oligosaccharides for anti-aging preparations	*M. agaves*	Isolate from the blue agave	Paufique (2013)
	*M. reukaufii* cells	Hydrolyzate with peptides for anti-aging preparations	*M. reukaufii*	Isolate from the nectar of *Hoya carnosa*	Paufique (2019)
	*M. andauensis* cells	Autolyzate with a higher level of hydroxyproline	*M. andauensis* D2	Isolate from the Polish apple	Pawlikowska et al. (2020)
	Glucose, L-phenylalanine (L-Phe)	2-phenylethanol (2-PE)	*M. pulcherrima* WUT8, *Metschnikowia* sp. WUT 12, 14, and 16	Isolates from Polish fruits	Chreptowicz et al. (2018)
	Glucose, L-Phe	2-PE	*M. pulcherrima* NCYC 373	Collection strain (NCYC, UK)	Chantasuban et al. (2018)
	Glucose, peptones	Pulcherrimin	*M. pulcherrima* CCY 145, CCY 149	Collection strains (CCY, Slovakia)	Kregiel et al. (2022a) and Kregiel et al. (2024)

### 4.1 Beverages

*Metschnikowia* is one of the most prevalent genera in grapevine phyllospheres, fruit flies, and grapes. This allows the use of some *Metschnikowia* species in winemaking (Table 1). Their ability to grow in association with other yeast species, such as *S. cerevisiae* or *Lachancea thermotolerans*, especially during the initial stages of wine fermentation, is also essential, modulating the synthesis of secondary metabolites. *Metschnikowia* yeasts have the potential to shape wine aromas and colors, and they can also be considered a tool for reducing ethanol content in wines (Hranilovic et al., 2020; Gonzalez et al., 2021). Reduction of both alcohol and acetic acid levels is achieved by controlling partial glucose respiration using a strain of *M. pulcherrima* (Guindal et al., 2023).

*Metschnikowia* spp. exhibit a moderate fermentative profile, but they show wide enzymatic activity, leading to the creation of flavor and color precursors. The combination of *Metschnikowia* and *S. cerevisiae* strains reduces alcohol production in wines, while imparting a fruitier and fresher aroma (Ruiz et al., 2018; Boscaino et al., 2019). Fermentations with *L. thermotolerans* and *M. pulcherrima* can increase acidity and modify the volatile profile of wines, imparting a fresher character. Escott et al. (2022) documented that the participation of these strains positively affected not only the volatile composition of wines but also color expression and consumer perception.

The key element here is the selection of yeast strains that may have a significant impact on wine quality (Torres-Díaz et al., 2025). *M. pulcherrima* strains are recommended in grape wine making for their contribution to the aromatic development of wine through their broad enzymatic activity (β-D-glucosidase, cysteine β-lyase) (Vicente et al., 2020) and the production of a wide range of esters and higher alcohols. This phenomenon was also observed in the case of fermentation of apple and apple-chokeberry musts (Kregiel et al., 2022b). The presence of *Metschnikowia* strains does not affect fermentation time, but reduces the fermentation rate of *S. cerevisiae*. Analysis of central carbon metabolism and volatile organic compounds reveals strain-dependent increases in metabolite production, including glycerol, acetate esters, medium-chain fatty acids, and ethyl esters (Aragno et al., 2024).

The results of numerous studies also suggest the potential of *Metschnikowia* species for bioprotection in wine production and quality (Kregiel et al., 2022b; Binati et al., 2023; Lebleux et al., 2023; Aragno et al., 2024). *Metschnikowia* spp. can be used as a stabilizer instead of SO₂ to obtain wines with low alcohol content and balanced color. It is possible to get wine stabilization with low SO₂ content and increase the content of aromatic substances such as ethyl butyrate and ethyl hexanoate (Canonico et al., 2023). The mixed cultures of *Metschnikowia* spp. and *S. cerevisiae,* and also *Metschnikowia* spp. and *L. thermotolerans,* act synergistically in the wine acidification process and may be used to improve sensory properties of grape and fruit beverages (Varela et al., 2021; Escott et al., 2022; Naselli et al., 2023; Kregiel et al., 2022b; Guo et al., 2025; Wu et al., 2025).

Recently, *M. pulcherrima* has also been used to ferment honey. The resulting beverages were characterized by lower ethanol content, higher glycerol level, and rich volatilomes. It is also worth noting that *M. pulcherrima* showed the highest tolerance to 30% w/v glucose (Kregiel et al., 2025).

Studies have shown that most *Metschnikowia* strains may be used in the brewery industry. These yeasts produce beer with an alcohol content of <0.5%. Higher β-glucosidase activity of *Metschnikowia* has a positive effect on beers (Klimczak et al., 2024).

Also other enzymes produced by *Metschnikowia* spp. may be particularly attractive in the beverage industry. De Souza et al. (2023) investigated the ability of *M. australis* to produce extracellular proteases at low temperatures. Yuivar et al. (2019) confirmed the yeast’s ability to produce extracellular gelatinase. Recent studies have demonstrated that *M. koreensis* can produce pectinases and proteases, which have numerous potential applications in the beverage industry (Theron et al., 2017; Snyman et al., 2019). These enzymes can regulate acidity, enhance aroma and clarity, and improve the color of fermented products (Wu et al., 2025). Pectinase has a significant impact on wine quality and clarity (Longhi et al., 2022). Furthermore, protease produced by *M. pulcherrima* can be used in American India Pale Ale (IPA) beer (Karaulli et al., 2024).

### 4.2 Cosmetics

The biological activity of *Metschnikowia* spp. also supports the use of their metabolites as active ingredients in cosmetic products (Table 1). Products derived from *Metschnikowia* species could reveal additional skin barrier-related attributes, because their secondary metabolites play a beneficial role in skin health (Mim et al., 2024).

The cosmetics industry mainly utilizes two species for skin care: *M. agaves* and *M. reukaufii*. *M. reukaufii* extracts contain various bioactive components, including peptides that have a beneficial effect on the skin microbiota (Paufique, 2019). In turn, *M. agav*es isolated from the blue agave of Mexico, and its hydrolase complex with α-glucan oligosaccharides and β-glucan oligosaccharides, naturally increases the production of hyaluronic acid, resulting in anti-aging, hydrating, and anti-wrinkle characteristics (Paufique, 2013).

Interestingly, Pawlikowska et al. (2020) studied the amino acid profiles of *Metschnikowia* spp. autolyzates. They produced biopreparations with a five-fold higher content of hydroxyproline, the main component of collagen. Therefore, such lysates could be used in the cosmetic industry as regenerating, revitalizing, smoothing, and moisturizing agents.

*Metschnikowia* spp. can produce 2-phenylethanol (2-PE). This compound is an aroma molecule primarily used in perfumes. Currently, the main method for producing this biobased compound is the extraction of trace amounts from rose petals, which is extremely expensive. However, this metabolite can be produced by *Metschnikowia* spp. and other yeasts using bioconversion processes. *Saccharomyces*, *Kluyveromyces, Pichia*, or *Metschnikowia* species can synthesize 2-PE by the biotransformation of L-phenylalanine (L-Phe) through the Ehrlich pathway. The productivity of 2-PE is associated with the type of yeast, carbon source, and media components (Mierzejewska et al., 2017). Chantasuban et al. (2018) developed a method for producing 2-PE using the *M. pulcherrima* strain in both batch and continuous modes. Other studies documented that 2-PE production varies depending on the kind of strain, medium composition, and fermentation conditions (Chreptowicz et al., 2018; Mitri et al., 2022).

Kregiel and co-workers found that *M. pulcherrima* strains can inhibit the growth of *Candida*- and *Candida*-related yeasts. This could be very useful for limiting the development of skin pathogens (Kregiel et al., 2023).

The interesting compound with great application potential in cosmetics is pulcherrimin—a red extracellular pigment formed by *Metschnikowia* spp. after growth in media enriched in iron (III). Freimoser and co-workers documented that pulcherrimin—an iron chelate of pulcherriminic acid—plays an essential environmental role in antagonistic microbial interactions, as well as in stress responses (Freimoser et al., 2019, 2024). Kregiel and co-workers investigated the biological activity of pulcherrimin produced by the *M. pulcherrima* clade. It was noted that this compound does not have antimicrobial properties. Still, its unique hydrophilic nature and Sun Protection Factor (SPF) may lead to interest in yeast pulcherrimin as an ingredient in moisturizing cosmetics with sun protection properties (Kregiel et al., 2022a; Kregiel et al., 2024).

### 4.3 Probiotics

*Metschnikowia* species may be also considered probiotic microorganisms (Table 2). Probiotics are live microorganisms that, when consumed in sufficient amounts, provide health benefits to the host (Chen et al., 2025). They have traditionally been used in dairy product technologies. Probiotic microorganisms improve people’s health and wellness, and research on this topic is relevant and interesting. Unlike probiotic bacteria, probiotic yeasts are relatively understudied. *Saccharomyces boulardii* is a patented probiotic yeast with functionality demonstrated in many studies (O’Brien et al., 2024; Kaźmierczak-Siedlecka et al., 2020). Although *S. boulardii* is the most well-characterized yeast available on the market, improving probiotic function using other yeast species is an attractive future direction for research. Some yeast strains of *M. ziziphicola* show interesting probiotic characteristics (Agarbati et al., 2020, 2024a,b; Staniszewski and Kordowska-Wiater, 2023). However, it was documented that the probiotic abilities are strictly strain-dependent. Results obtained by Smith et al. (2015) demonstrated that *M. gruessii* can protect human epithelial cells from invasion by *Salmonella enterica* subsp. *enterica* serovar Typhimurium. In addition, a recent study conducted by Rodríguez Machado et al. (2024) showed that *M. chrysoperlae* strains may also be considered as probiotic agents. These yeast strains could be proposed for various probiotic applications, offering a valid alternative to or in combination with the probiotic yeast *S. boulardii* (Agarbati et al., 2020).

**Table 2:** Possible applications of *Metschnikowia* spp. to inhibit pathogenic microorganisms.

Possible application	*Metschnikowia* species	Origin	Antagonistic activity against	References
Probiotics	*M. ziziphicola* B27	Isolate from beech tree bark	*Candida albicans* *Escherichia coli* *Listeria monocytogenes* *Staphylococcus aureus* *Salmonella enterica*	Agarbati et al. (2020)
	*M. ziziphicola* Mz82	Isolate from beebread	*L. monocytogenes* *S. enterica*	Agarbati et al. (2024b)
	*M. chrysoperlae*	Isolate from Chilean Quillay honey	*E. coli* ATCC 35218™ *S. aureus* ATCC BAA-1026™ *S. enteritidis* ATCC 49223™	Rodríguez Machado et al. (2024)
Biocontrol	*M. pulcherrima* 02.11.1.21 02.4.3.38	Isolates from botrytized grapes	*Botritis cinerea* 3318	Sipiczki (2006)
	*M. pulcherrima* NCYC747 *M. andauensis* D2, D4, D5, D7, D8 *M. sinensis* D1, D3, D9, D10	Collection strain (NCYC, UK) and isolates from Polish flowers and fruits	*Alernaria alternata* LOCK409 *B. cinerea* LOCK453 *Penicillium expansum* LOCK535 *Verticillium cinnabarinum* LOCK576 *Wickerhamomyces anomalus* C1 (NCYC D5299) *Dekkera bruxellensis* C2 (NCYC D5300)	Pawlikowska et al. (2019)
	*M. citriensis* FL01	Isolate from healthy citrus leaves	*Geotrichum citri-aurantii* (isolated from infected citrus fruit)	Wang et al. (2020), Wang et al. (2022)
	*M. pulcherrima* APC1.2	Isolate from apple flowers	*Gibberella* (*Fusarium*) *fujikuroi* BC 8.14, CCOS1020, SH213620.07FU (isolated from soil)	Bühlmann et al. (2021)
	*M. fructicola*	Isolate from fruits	*P. digitatum* NRRL1202 *P. expansum* DSM6284	Oztekin and Karbancioglu-Guler (2021)
	*M. pulcherrima* TK1	Isolate from the strawberry flower	*Fusarium sambucinum* DSM 62397 *Rhizoctonia solani* DSM 22843, *A. tenuissima* DSM 63360	Steglińska et al. (2022)
	*M. pulcherrima* Mp-22 Mp-30	Isolates from *Vitis vinifera* (cv. Grenache)	*A. alternata* CECT 20560 *B. cinerea* CECT 20754	Fernandez-San Millan et al. (2022)
	*M. pulcherrima* MPR3 *M. fructicola* NRRL Y-27328	Collection strains (Di3A, Catania) commercial strain (NOLI, Italy)	*Erysiphe necator* *B. cinerea*	Lombardo et al. (2023)
	*M. citriensis* FL01	Isolate from leaves (Liu et al., 2018)	*P. italicum* *G. citri-aurantii* *P. digitatum*	Zhang et al. (2023)
	*M. rancensis*	Isolate from blueberry flowers	*Colletotrichum acutatum* *C. fioriniae* *C. gloeosporioides*	Rering et al. (2023)
	*M. pulcherrima* CLIB 3131 CLIB 3132 CLIB 3139 MTF 4325	Isolates from grape musts	*Brettanomyces bruxellensis* *Gluconobacter oxydans* (isolated from grapes and wine)	Aragno et al. (2024)
	*M. persimmonensis* KIOM G15050	Isolate from *Diospyros kaki* calyx	*F. oxysporum* *B. cinerea*	Rahmat et al. (2024)
	*M. pulcherrima* 62, 86, AS3C1	Collection strains (Di. A. A. A., Italy)	*Ascosphaera apis* (isolated from *A. mellifera* larvae)	Iorizzo et al. (2025)
	*M. pulcherrima* WM05	Isolate from the grape peel	*P. digitatum* *P. italicum* *G. citri-aurantii* (isolated from surfaces of naturally infected citrus fruits)	Liu et al. (2025)

The probiotic nature of yeasts is usually defined by resistance to low pH, survival and growth capacity at high temperatures (37 °C), tolerance to gastric acidity, resistance to bile salts, auto-aggregation capability, and resistance to the gastrointestinal tract. These are the most commonly used criteria for selecting probiotic strains to balance the intestinal microbiome. Rodríguez Machado et al. (2024) demonstrated that it is possible to isolate yeasts with potential probiotic characteristics from honey. The yeast *M. chrysoperlae* was shown to be able to tolerate low pH, bile salts, and temperatures of 37 °C in a simulated *in vitro* digestion system, maintaining cell concentrations above 10^6^ CFU/mL. The yeasts can also auto-aggregate and show capabilities for controlling enteric pathogenic bacteria. Findings obtained by Agarbati et al. (2020) showed interesting probiotic characteristics for some non-conventional yeast isolates belonging to *M. ziziphicola* that inhibited the growth of both bacterial and fungal pathogens. Subsequent studies by Agarbati et al. (2024b) confirmed the suitability of *M. ziziphicola*, originating from honeybee ecosystems, to inhibit both *Listeria monocytogenes* and *S. enterica*. These findings encouraged future efforts aimed at confirming the observed effects *in vivo* and driving further strain development toward novel yeast probiotics.

### 4.4 Biocontrol

Diverse antagonistic properties of *Metschnikowia* strains provide a basis for the use of active strains as biocontrol agents. The antimicrobial nature of *Metschnikowia* species has been confirmed mainly for filamentous fungi and some yeasts (Table 2). Growth inhibition by *Metschnikowia* spp. was demonstrated against prevalent pathogens, including *Alternaria alternata* (Pawlikowska et al., 2019; Fernandez-San Millan et al., 2022), *Botritis cinerera* (Sipiczki, 2006; Fernandez-San Millan et al., 2022), *Penicillium* spp. (Pawlikowska et al., 2019; Oztekin and Karbancioglu-Guler, 2021; Liu et al., 2025), *Geotrichum citri-auranti* (Wang et al., 2020, 2022; Zhang et al., 2023) and *Colleotrichum* spp. (Rering et al., 2023). Also noteworthy is the activity of *Metschnikowia* spp. against *Fusarium oxysporum* and *Gibberella (Fusarium) fujikuroi* (Rahmat et al., 2024; Bühlmann et al., 2021), which are widespread worldwide and cause diseases called fusariosis. Antimicrobial activity of *Metschnikowia* species was also reported against more specific pathogens, e.g., *Erysiphe nectator* (Lombardo et al., 2023), which causes powdery mildew of grapevines, and *Ascosphera apis* (Iorizzo et al., 2025), which exclusively infects honeybee larvae.

For the biocontrol yeasts, multiple mechanisms such as competition for nutrients and space, secretion of enzymes, toxin production, formation of volatile organic compounds (VOCs), mycoparasitism, and induction of resistance in plants are likely to be involved in the antagonistic function. However, in most cases, the mechanisms outlined and discussed below have not been fully proven by molecular analyses (e.g., by gene deletion and complementation, heterologous expression), but instead proposed based on analogies with other biological systems (Freimoser et al., 2019).

Pulcherrimin formation and iron depletion are the main mechanisms by which *Metschnikowia* exerts biocontrol effects (Rahmat et al., 2024; Sipiczki, 2006, 2020; Pawlikowska et al., 2019). As the reaction of pulcherriminic acid with ferric ions is irreversible and pulcherrimin is insoluble in water, the process is unlikely to play a role in iron acquisition. The chelated iron in pulcherrimin is inaccessible to the biochemical processes of microorganisms. Since iron is required for the activity of many proteins and cellular processes, its immobilization by pulcherriminic acid adversely affects the propagation of many microorganisms. Due to antimicrobial properties, pulcherrimin-producing *Metschnikowia* strains can be utilized as biological agents to protect agricultural commodities and food products against pathogenic and destructive microorganisms (He et al., 2024).

Pulcherrimin formation may be variable and reversible (Sipiczki et al., 2024). Interestingly, pulcherrimin production also varied within species. Intraclonal changes (segregation) in the intensity of pulcherrimin production in *M. pulcherrima* were reported. The cultures formed sectors differing in color intensity and mixtures of differently colored colonies. It was assumed that these changes may be due to different processes, e.g., silencing and reactivation of regulators, as well as mutations and backmutations, but this was not clearly explained.

The pulcherrimin biosynthesis was studied by Larini et al. (2025), who extracted gene sequences responsible for proteins related to pulcherriminic acid production and transport (*PUL1, PUL2, PUL3,* and *PUL4*). Then, the analysis of the flanking regions was conducted to understand the genetic configuration and explain the reversible character of pulcherrimin formation. In almost all strains tested, at least two copies of each *PUL* gene were found per strain, but an exception was *M. pulcherrima* KIOM G15050 (formerly *M. persimmonensis*). Most genomes exhibited *PUL* genes arranged in the order *PUL1-PUL2-PUL4-PUL3*; however, this was not a universal rule. *M. pulcherrima* strain NRRL Y-7111 T had syntenic *PUL1, PUL2*, and *PUL4*, but another copy of *PUL4* and *PUL3* was localized to a different contig. In another strain, *M. pulcherrima* 277, the *PUL*3 gene was located between the *PUL2* and *PUL4* genes. The prediction of protein localization revealed that proteins PUL1 and PUL4 appear to be located in the cytoplasm or nucleus, PUL2 is in the endoplasmic reticulum, PUL3 is in the cell membrane, and SNF2, a transcriptional regulator involved in pulcherrimin production, is in the nucleus for all strains. The predicted localizations of these proteins reflected their functions, as PUL1 and PUL2 are involved in pulcherriminic acid synthesis, PUL3 appears to be a transporter, and PUL4 and SNF2 are transcription factors that regulate the biosynthesis process.

In the context of biocontrol, carbohydrate-active enzymes (CAZymes) are also of particular interest. Their activity reflects the strain’s ability to colonize plant surfaces and its potential as a biocontrol agent. Strains are specific to plant surfaces, especially for the glycoside hydrolases. Enzymes can also participate in fungal cell wall degradation. Larini et al. (2025) studied CAZymes for which a signal peptide was predicted, indicating a putative extracellular location. Signal peptides play a key role in protein secretion, making them particularly interesting for the development of biological control agents (Thak et al., 2020). For example, Jones and Prusky (2002) demonstrated the potential for expressing antifungal peptides in yeast, which represents a novel approach to post-harvest disease control.

Chitinase activity may also contribute to the antagonistic effect of *M. pulcherrima* (Minguet-Lobato et al., 2024). Banani et al. (2015) observed that the *M. fructicola* AP47 strain showed higher transcription intensity of the chitinase gene in the presence of the *Monilinia fructicola* fungus. Antagonistic testing studies have shown that *Metschnikowia* spp. exhibits chitinase activity, while other strains exhibit protease, pectinase, and cellulase activity. These enzymatic actions can destroy the surface structures of fungal pathogens, enhancing their inhibitory effects (Acar et al., 2024).

Volatile organic compounds (VOCs) produced by *Metschnikowia* can also act on the pathogen directly and exert resistance. Studies conducted by Vepštaitė-Monstavičė et al. (2025) and Steglińska et al. (2023) confirmed the important correlation between antimicrobial action and VOCs produced by *Metschnikowia*, highlighting their role in ensuring antagonistic efficacy. The most abundant VOCs produced by *Metschnikowia* are esters and alcohols. Among these, the most abundant esters are ethyl acetate and 3-methylbutyl acetate. In turn, the most abundant alcohols are 2-phenylethanol and ethanol.

Switching from the planktonic yeast growth to the formation of chains of non-separated cells (pseudohyphae) may play an essential role in the protection of the plant surface because the pseudomycelium formed by the invasive pseudohyphae can form biofilms on the lesions, which are also gateways for the invasion by destructive microorganisms (Sipiczki et al., 2024). The efficiency of the yeast-to-pseudomycelium transition and the morphology of substrate invasion may greatly vary among the strains (Lachance, 2011). Laboratory studies examined the ability of *Metschnikowia* planktonic cells to adhere to non-plant-defined surfaces, including glass (hydrophilic) and polypropylene (hydrophobic) (Pawlikowska et al., 2019). Sipiczki et al. (2024) examined the ability of the tested strains to form pseudohyphae. Because a pseudomycelium is a stronger structure than a layer of planktonic yeast cells, especially if its pseudohyphae establish a strong bond with the damaged plant tissue by penetrating it. The efficiency of the yeast-to-pseudohyphal transition and the morphology of substrate invasion by the pseudomycelium also varied significantly among the isolates.

According to the FDA, strains of *M. pulcherrima* and *M. fructicola* may be used post-harvest, individually or in combination, at a maximum level of 1 g yeast/kg fresh coffee cherries, providing up to 2 × 10^7^ CFU/g coffee (FDA, 2022). Some strains of *Metschnikowia* spp. are active against *P. expansum* and can be used for apple protection (Acar et al., 2024; Settier-Ramírez et al., 2021). Others are active against *B. cinerea* and can be used for protection against grape or apple diseases (Altieri et al., 2023). Initial experiments have demonstrated the ability of *M. pulcherrima* to inhibit the growth of undesired microorganisms in horticultural plants (potato seeds and strawberries) during post-harvest processing (Steglińska et al., 2022; Pawlikowska et al., 2019). Steglińska et al. (2023) reported a favorable interaction between *Metschnikowia* spp. and garlic for biological control. In other studies, *Metschnikowia* strains have been used as a potential natural biocontrol agent for stored fruits, including lemons, apples, grapes, sweet cherries, strawberries, and mangoes (Oztekin and Karbancioglu-Guler, 2021; Tian et al., 2017; Fernandez-San Millan et al., 2022).

## 5 Bioconversion as a primary tool in green technology

Currently, *Metschnikowia* spp. are being explored as promising agents for environmentally friendly and cost-effective green technologies. Biotransformation using waste is mainly used to produce lipids, proteins, enzymes, and biofuels. Table 3 shows many potential bioconversions of various by-products by *Metschnikowia* species.

**Table 3:** Potential of *Metschnikowia* spp. for green technology.

Type of waste	*Metschnikowia* species	Origin	Product(s)	Limitations	References
Lignocellulosic biomass (wood, straw) hydrolysate	*M. pulcherrima* 11	No data	Lipids	Inhibitors limit yeast growth and oil formation	Zhou et al. (2017)
Crude glycerol	*Metschnikowia* sp. P. D.-F1 P. D.-D2 V. V.-D4	No data	Lipids with oleic and palmitic acids	Experiments on a laboratory scale	Diamantopoulou et al. (2020)
Lignocellulosic biomass (food waste, rice straw, softwood sawdust) hydrolysate	*M. pulcherrima* CHN-FW374	Isolate from rotten food wastes	Lipids 2-PE	Need for pre-treatment	Abomohra et al. (2021)
Crude animal fat	*M. pulcherrima* CCY 29-02-145 CCY 29-02-147 CCY 29-02-14 CBS 5833 (11-1235) *M. andauensis* CCY 29-02-12 HA 1657 (11-1241) *M. chrysoperlae* CBS 9803 (11-1158) *M. fructicola* CBS 8853 (11-1235) *M. sinensis* CBS 10357 12	Collection strains (CCY, Slovakia; CBS, Netherlands)	Polyunsaturated fatty acids	Need for substrate emulsification	Němcová et al. (2021)
Persimmon pomace	*M. pulcherrima* M7	Isolate from grape must	Lipids	Lower yield compared to other oil yeasts	Tatay-Núñez et al. (2024)
Whey, by-products from sugar beet processing	*M. chrysoperlae* WUT25	Isolate from green grapes, Armenia	2-PE	Lower yield compared to *S. cerevisiae*; need for L-Phe supplementation	Chreptowicz et al. (2018)
Pomegranate peel, Melon peels Mango pomace	*M. cibodasensis* Y34	Isolate from *Abelia* flowers	Ethanol	Need for pre-treatment and substrate detoxification	Chaudhary et al. (2021a) Chaudhary et al. (2021b) Chaudhary et al. (2022) Chaudhary et al. (2024)
Whey after lactose hydrolysis	*M. pulcherrima* E1	Isolate from loquat leaves	D-arabitol, L-galactitol	Two-step process, long fermentation	Zhang et al. (2022)
Tofu whey	*M. pulcherrima* Flavia	Commercial strain (PROENOL, Portugal)	Ethanol (low alcoholic beverage)	Trace levels of product	Chua et al. (2018)
Soy pulp (Okara)	*M. pulcherrima* Flavia	Commercial strain (Lallemand)	Aroma compounds	Lower yield compared to *Williopsis saturnus*	Vong and Liu (2017)
Sugar beet pulp Repeseed meal	*M. pulcherrima* NCYC 747	Collection strain (NCYC, UK)	Single-cell proteins (SCP)	Need for pre-treatment of the substrate; lower yield compared to fodder yeasts	Dygas et al. (2023a) Dygas et al. (2023b)

Possible raw materials for bioconversion processes involving *Metschnikowia* spp. included fruit wastes (Chaudhary et al., 2021a, 2021b, 2022, 2024; Tatay-Núñez et al., 2024) and various lignocellulosic biomass (Vong and Liu, 2017; Zhou et al., 2017; Abomohra et al., 2021; Dygas et al., 2023a, 2023b). Potential substrates also involved whey (Chreptowicz et al., 2018; Zhang et al., 2022; Chua et al., 2018), and even post-production animal fats and glycerol (Němcová et al., 2021; Diamantopoulou et al., 2020).

In this context, lipid production seems to be particularly interesting. The interest of biotechnologists is closely linked to the rising prices of fossil fuels. Their negative impact on the environment is undeniable. The composition of microbial lipids is very similar to that of vegetable oils, which creates significant potential for their use in biodiesel production. This biotechnological strategy could also be interesting for the food industry (Abeln and Chuck, 2019; Abeln et al., 2019; Abeln et al., 2020).

Traditionally, economical and environmentally friendly production is achieved by metabolizing nutrients produced by algae. This method, which recycles solid waste such as macroalgae sugars and proteins, holds great potential for sustainable economic development. Li et al. (2021a) achieved a significant reduction in harmful substrate emissions from organic waste, alongside the production of *Metschnikowia* oil. It is worth noting that *Metschnikowia* spp. can utilize acetic acid from organic waste to enhance lipid accumulation (Li et al., 2021b).

Němcová et al. (2021) demonstrated the ability of yeast to produce significant amounts of unsaturated fatty acids from crude waste animal fat, with the accumulated lipids in yeast cells reaching 36% of cell dry weight. It was documented that controlling nitrogen content can increase lipid content compared to phosphorus-limited conditions.

Diamantopoulou et al. (2020) cultivated yeasts of the genus *Metschnikowia*, among others, under nitrogen-limited conditions using crude glycerol as a substrate. Lipids produced by yeasts contained mainly oleic and palmitic acids. However, *Metschnikowia* strain produced lower amounts of lipids than the best strain, *Rhodosporidium toruloides*, which was able to form oil from glycerol in an amount of 12.5 g/L.

In a study conducted by Tatay-Núñez et al. (2024), *M. pulcherrima* grew well in sugarcane and sugar beet molasses, as well as persimmon hydrolysate, and produced intracellular lipids. Their cells demonstrated increased tolerance to desiccation. However, the lipid yield was lower in comparison to other tested oleaginous yeasts.

*Metschnikowia* strains are also able to grow on lignocellulosic biomass for lipid production (Zhou et al., 2017; Abomohra et al., 2021). In the work of Zhou et al. (2017), rapid, microwave-assisted acidolysis of lignocellulosic biomass led to the production of fermentable saccharides. Under these conditions, 1.5 g/L of mono- and di saccharides were available for fermentation, and less than 0.5 g/L of acids and furfurals were produced, which prevented fermentation by *S. cerevisiae* and other ethanol-producing yeasts. *M. pulcherrima* could grow in this broth, producing small amounts of lipids with a composition similar to palm oil.

The valorization of food and lignocellulosic wastes into biodiesel using *M. pulcherrima* strain isolated from rotten food wastes was evaluated by Abomohra et al. (2021). Food waste hydrolysate was supplemented by rice straw and softwood sawdust as additional carbon sources to increase the C: N ratio. *M. pulcherrima* showed the ability to produce lipids with a maximum productivity of 2.49 g per liter per day, and good biodiesel characteristics.

Intensive research into fermentation technology for waste biomass revalorization resulted in a study of *M. pulcherrima* as a source of SCP production. In a study by Dygas et al. (2023b), *M. pulcherrima* was successfully grown on rapeseed meal. A slight increase in protein was obtained, measured by an increase in nitrogen content from 0.6 to 1.6%. However, simultaneous saccharification and fermentation led to the conversion of isoflavones into forms with fewer adverse effects and lower estrogenic activity. In other work, the same authors investigated the possible use of *M. pulcherrima* to enrich sugar beet pulp pretreated by enzymatic hydrolysis. In these conditions, *Metschnikowia* yeast can grow at the level 2 × 10^7^–1 × 10^8^ CFU/mL, and the protein increase measured by the increase in nitrogen content ranged from 0.9 to 1.7% (Dygas et al., 2023a). The results showed that sugar beet pulp provides a good matrix for SCP and feed production. However, when using lignocellulosic biomass, pre-treatment is necessary.

The production of bioethanol and other alcohols from agricultural byproducts offers a good solution for waste management. *Metschnikowia* strains were used for this purpose, even though they are moderate fermenting yeasts. Research on the use of *Metschnikowia* spp. in fermentation processes was conducted on various lignocellulosic wastes. Despite wide yeast enzymatic activity, lignocellulosic biomass pretreatment was necessary (Chaudhary et al., 2021a,b; Chaudhary et al., 2022; Chaudhary et al., 2024). However, the drastic thermal and chemical treatment of lignocellulosic biomass means these bioconversions cannot be classified as pure green technologies.

Chreptowicz et al. (2018) assessed the activity of *M. chrysoperlae* in the production of 2-phenylethanol from whey or molasses, but the results also were not satisfactory. *Metschnikowia* strain produced 2-PE at 1 g per liter in whey and sugar beet juice medium; however, the best producers were strains from *S. cerevisiae*, which produced about 3 g/L of this metabolite.

Mitri et al. (2022) described different weak points for 2-PE formation. Firstly, high concentrations of 2-PE inhibit the yeast cell growth. Of course, there are several ways to overcome the inhibiting restraint. For example, strain mutagenesis and culture medium composition, or the optimization of the fermentation conditions, are widely used strategies for the improvement of yield. Secondly, the nitrogen sources, including L-Phe, carbon sources, vitamins, and minerals, supplemented to the media affect the fermentation process. For this reason, these media are not cost-effective, and it is necessary to search not only for efficient strains but also for various alternative, cheap cultivation media. In addition, temperature, initial medium pH, and fermentation time are all factors that could affect the amount of product.

Dairy industry wastes can be explored as a cheap and attractive raw material also for producing various alcohols. Zhang et al. (2022) converted cheese whey powder into D-arabitol and L-galactitol in a two-step process. Firstly, the simultaneous lactose hydrolysis and isomerization of lactose-derived D-galactose was performed by an engineered *E. coli* strain. Subsequently, the mixture containing lactose-derived D-glucose and residual D-galactose was subjected to fermentation by *M. pulcherrima* E1 strain, which produced 60 g/L D-arabitol and 28 g/L galactitol, low-calorie sweeteners.

*Metschnikowia* spp. are a promising microorganism with huge biotechnological potential. However, so far, these biotechnological strategies are still not economically competitive with chemical synthesis. To sum up the examples of the use of waste materials by the *Metschnikowia* spp., it should be stated that the first ambitious goal for developing biotechnological production should be to identify highly productive yeast strains and substrates that can be revalorized. The task will also be to use appropriately selected mixed cultures, proper pre-treatment methods, and cultivation conditions.

## 6 Conclusion

**Figure 3:**
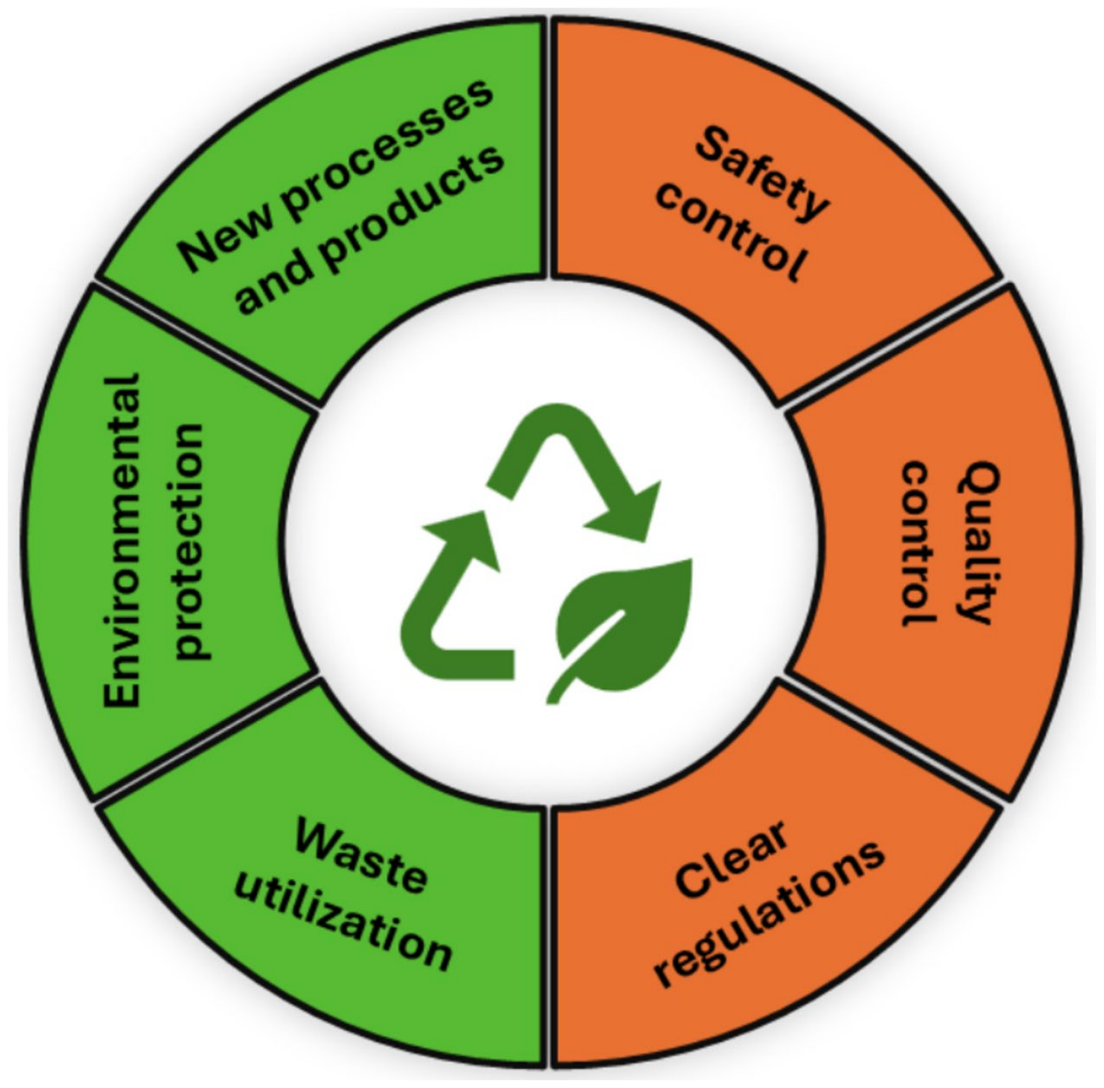
*Metschnikowia* spp. in green technologies—potentialities (green) and limitations (orange).

The main standards of green technology focus on reducing environmental pollution and reusing waste to produce new products (Nandy et al., 2022). Green technologies offer numerous opportunities, but they also face significant challenges (Figure 3). Green innovations can leverage new ideas to develop new processes and products, as well as improve existing production processes through environmentally friendly practices. Therefore, implementing closed-loop green technologies is essential for a sustainable future on a global scale. Current research is focused on developing new industrial methods and technologies that not only reduce waste and dependence on raw materials but also promote ecosystem conservation.

The use of *Metschnikowia* spp. in green processes offers several advantages, such as utilizing their broad enzymatic capabilities, non-toxicity to humans and the environment, low cost, and long-lasting protection against pathogens. With the continuous advancement of green technologies, scientists are increasingly exploring the use of waste materials for culturing *Metschnikowia* cells, with broader applications in sectors such as food/feed, energy, cosmetics, and biocontrol. However, it is noteworthy that the long-term use of *Metschnikowia* in various natural environments can impact other beneficial microorganisms and disrupt microbial ecological systems (Nowak et al., 2025).

Several regulations govern the use of *Metschnikowia* spp. in the food/feed industry. According to these guidelines, particular attention should be paid to the safety of the yeast strain, human- and environmentally friendly process conditions, and the economic viability of such processes in large-scale industrial production. Therefore, to enable the full commercialization of *Metschnikowia*-based products, it is essential to establish quality standards and specifications that ensure safety, efficacy, and consistency. First, standards for raw materials should specify the required purity for yeast cell production. Additionally, process standards should be developed, covering fermentation conditions, hygiene protocols, and methods for detecting all potentially toxic substances. Both the stability of the innovative products and other key parameters must be clearly defined and implemented.
